# 

**Published:** 1886-03

**Authors:** 


### CONTRIBUTORS.

| | |
|---|---|
| Joseph Eve Allen, M. D. | Atlanta, Ga. |
| Robert Battey, M. D. | Rome, Ga. |
| L. E. Borcheim, M. D. | Atlanta, Ga. |
| A. W. Calhhoun, M. D. | Atlanta, Ga. |
| W. Cheatham, M. D. | Louisville, Ky. |
| Hunter P. Cooper, M. D. | Atlanta, Ga. |
| R. O. Cotter, M. D. | Macon, Ga. |
| Joseph A. Eve, M. D. | Augusta, Ga. |
| A. Freeman, M. D. | New York, N. Y. |
| James A. Gray, M. D. | Atlanta, Ga. |
| J. Edward Green, M. D. | Macon, Ga. |
| Virgil O. Hardon, M. D. | Atlanta, Ga. |
| A. G. Haygood, D. D. LL.D. | Oxford, Ga. |
| Jno. Hockenhull, M. D. | Cumming, Ga. |
| T. M. Holmes, M. D. | Rome, Ga. |
| Joseph Holt, M. D. | New Orleans, La. |
| J. M. Hull, M. D. | Augusta, Ga. |
| G. C. Hummel, M. D. | Savannah, Ga. |
| M. B. Hutchins, M. D. | Lawrenceville, Ga. |
| Harry Huzza, Esq. | Atlanta, Ga. |
| Jno. Thad Johnson, M. D. | Atlanta, Ga. |
| Seth N. Jordan, M. D. | Columbus, Ga. |
| N. B. Kennedy, M. D. | Hillsboro, Tex. |
| J. C. Le Hardy, M. D. | Savannah, Ga. |
| V. D. Lockhart, M. D. | Homer, Ga. |
| Joseph P. Logan, M. D. | Atlanta, Ga. |
| H. S. Lott, M. D. | Augusta, Ga. |
| H. HcHatton, M. D. | Macon, Ga, |
| E. Miller, M. D. | Florence, S. C. |
| H. V. M. Miller, M. D. LL.D. | Atlanta, Ga. |
| K. P. Moore, M. D. | Macon, Ga. |
| R. J. Nunn, M. D. | Savannnah, Ga. |
| W. O'Daniel, M. D. | Bullard's, Ga. |
| R. St. J. Perry, M. D. | Cape Mount, Africa. |
| S. Latimer Phillips, M. D. | Savannah, Ga. |
| J. W. Russey, M. D. | Rising Fawn, Ga. |
| H. H. Smith, M. D. | Philadelphia, Penn. |
| Lawson Tait, F. R. C. S. | Birmingham, Eng. |
| V. H. Taliaferro, M. D. | Atlanta, Ga. |
| David Webster, M. D. | New York, N. Y. |
| Geo. R. West, M. D. | Rome, Ga. |
| W. F. Westmoreland, M. D. | Atlanta, Ga. |
| Henry Wile, M. D. | Atlanta, Ga |

### SOCIETIES.

Atlanta Society of Medicine.
Baltimore Academy of Medicine.
Chicago Medical Society.
Clinical Society of Maryland.
Macon Medical Society.

### INDEX.

### A

Advice to young doctors 72.
A horrible traffic 444.
Alcoholic liquors. The relation of the profession to the use and abuse of 273, 520.
An Anatomical Act for Georgia 579.
An Anatomical bill 642.
Anidrosis 488.
Atlanta, A long felt want in 445.
Report of Board of Health of 115.
Society of Medicine, annual dinner of 325.

### B

Bacteria, The methods of microscopical examination and artificial culture of 89, 154.
Battey's operation, A history of 657, 262, 375, 385, in Paris 777
Bodies of the dead, Protection for 639.
Br midrosis 489.
Books Received 383, 517, 780.
Book Reviews: Antiseptic surgery 514, A hand book on diseases of the nervous system 572, A laboratory guide on Urinalysis and Toxicology 635, Analysis of the Urine 634, A Compend of Pharmacy 443, A manual of Dietetics 574, A manual of Microscopical Techniology 50, A manual of Operative Surgery 47, A manual of Practical Therapeutics 577, A system of practical medicine 315, 515, A treatise on Electrolysis 575, A treatise on diseases of infancy and childhood 634, A treatise on diseases of the Nervous System 631, Bright's disease and allied sffections 440, Clinical notes on Uterine Surgery 49, Cutaneous memoranda 112, Climatology and mineral waters of the United States 322, Diseases of the lungs 112, Diagnosis of diseases of the brain and spinal cord 319, Dietetics and Dyspepsia 380, Diseases of the spinal cord 383, Diseases of the stomach and intestines 573, From Lincoln to Cleveland 51, Guide to the examination of Urine 50, Hand-book of general therapeutics 577, Hand-book of practical medicine 709, Illustrations of unconscious memory in disease 442, Insanity and its treatment 383, International Encyclopædia of Surgery 437, Local Anæthesia 381, Medical Communications of Massachusetts Medical Society 110, Medicine of the future 710, Operative Surgery of the Brain 380, Practical notes on skin diseases 51, Principles and Practice of Surgery 378, Paralysis Cerebral bulbar and spinal 632, Pathology and Treatment of Syphilis 637, Physicians Visiting List 710, Reference Hand-book of the Medical Sciences 187, 512, Rheumatism 576, The Genuine Works of Hippocrates, 441, The Methods of B cteriological Investigation 381, The Physician's Leisure Hour Series 516, The Student's Manual of Venereal Diseases 441, Transactions Texas State Medical Association 778.
Brain, Surgery of 599.

### C

Cæsarean section, a case of 21.
Calomel as a diuretic 42.
Carbolic acid as a hypodermic injection for the cure of hemorrhoids, carbuncles, etc. 289.
Cataract operations 29.
Chromidrosis 489,
ocaine, the other side of, the bad side 418, a defense of in cataract extractions 477.
Comedo 541.
Convicts, medical service for 640.
Coroner, the office of 580.
Correspondence: A case of faith cure 46, Augusta letter 183, a Georgia doctor in New York 627, 7[illegible]5, New York letter 256, 509, 569, 623, 701, 774, Philadelphia letter 43, 507, 564.
The proposed Congress of American Physicians and Surgeons, and how it will affect the American Medical Association 309.
County jails of Georgia 113.
Corset splint, 682.
Criminal abortion in Atlanta 191.
ross-eyes, a case of 603.

### D

Diagnosis, new departures in 53, 190.
Diphtheria, oil turpentine in 60.
Doctors, vacations for 390.
Diarrhœa or summer complaint 420.

### E

Earache 481.
Ear, acute suppuration of the middle 550
Eczema 675, erythematosum 676, fissure 678 papulosum 677, pustulosum 677, rubrum 677, Squamosum 678, vesiculosum 677, etiology 737, pathology 739, diagnosis 740, prognosis 743, treatment 743.
Erythema, nodosum 596, multiforme 594.
Eye and ear, syphilis of 289.
Eye, syphilis of 95.

### F

Faith Cure, A case of 46.
Fistula in perineo 548.
Fluid Extracts *vs* Tinctures, 745.
Fractures, The treatment of 80, of the Coracoid process of Scapula 161.

### H

Hematidrosis, 490.
Hemorrhoids and fissure of anus, treatment of 359.
Hyperæmia active 543, passive 545.
Hyperidrosis 485.

I

Issue of Veracity between Dr. Robert Battey and Mr. Lawson Tait 262, 375.
Items: 28, 32, 42, 58, 121, 171, 194, 255, 263, 314, 328, 390, 451, 521, 538, 549, 563, 582, 602, 605, 622, 647, 687, 689, 700, 719, 787.

K

Kidney Chronic Cirrhotic, A case of 495.

L

Liver, Surgery of 712.

M

Malaria and its toxic influences. Malarial hæmaturia 163.
Medical Association of Georgia 56, 201, 258, 120
Medical Colleges—Atlanta, Commencement of 119 - Southern, Commencement of 122.
Medical Congress. The International 518.
Medical Degree—A national and international 129.
Medicine, the progress of 529.
Medical profession of Georgia, organization of the 447.
Milium 542.

N

Neurasthenia 750.
New code man on newspaper puffs 312.
New-years Greeting 711.
Nymphæ—Hypertrophy of 606.

O

Obituary—Dr. M. J. Burt 489, Dr. J. J. Caldwell 129, Dr. J. B. Ficklin 127; Dr. Austin Flint, Sr. 118, Dr J. M. Johnson 261, 265, Dr. F. H. Hamilton 450.
Obstetrics and Gynecology—Clinical contributions to 1.
Osmidrosis 489.
Otalgia, 688.
Our advertisers 63, 200, 267, 335, 400, 463, 528, 592, 656, 723, 790.
Our Journal 52.

P

Polyclinic for Atlanta, 117.
Preservation of the Perineum during the expulsion of the fœtal head 410.
Phimosis with exaggerated symptoms 493.
Personal: Dr. J. W. Ainger 391, Dr. J. F. Alexander, 263, Dr. W. S. Armstrong 391, Dr. W. C. Ashley 647, Dr. A. B. Ashworth 264, Dr. J. C. Avary 390, 521, Dr. Robert Batey 647, 784, Dr. S. D. Brantley 262, Dr. C. A. Brooks 247, Dr. A. W. Calhoun 451, Dr. H. P. Cooper 329, 521, 582, Dr. R. O. Cotter 59, 521, Dr. Jno. W. Cox 59, Dr. W. C. Dabney 452, Dr. F. E. Daniel 452, Dr. K. H. Davis 263, Dr. J. A. Dunwody 647, Dr. W. S. Elkin 391, Dr. W. G. England, 719, Dr. J. E. English 329, Dr. Paul Faver, 521, Dr. J. McF. Gaston, 328, Dr. A. W. Griggs 58, Dr. V. O. Hardon 122, 329, Dr. N. O. Harris 523, 582, Dr. W. F. Holt 583, Dr J. G. Hopkins 328, Dr. D. H. Howell 58, 583, Dr. J. M. Hull, 328, Dr. G. C. Hummel 784, Dr. E. G. Janeway 194, Dr. Jno. Thad. Johnson 785, Dr. L. H. Jones 654, Dr. H. McHatton 329, Dr. T. M. McIntosh 525, Dr. P. C. Magnus 328, Dr. H. V. M. Miller 391, Dr K. P. Moore 583, 719, Dr. Stephens Neal 583, Dr. C. W. Nutting 264, Dr. F. H. Orme 399, Dr. R. C. Stockton Reed 264, Dr F. A Scribner 654, Dr. J. P. Taylor 328, Dr. R. H. Taylor 452, Dr. T. G. Thomas 196, Dr. C. I. Thompson 451, Dr. J. S. Todd 391, Dr. W. F. Westmoreland 263, Dr. W. F. Westmoreland, Jr. 194, 333, 451, Dr. H. Wile 266, 451, Dr. J. D. Wilson 583, Dr. A. J. Woodward 451, Dr. T. J Word, 328

S

Sarcoma Giant Cell, 761.
Sebaceous Cysts 542.
Seborrhœa 591, 539.
Should preachers pay 685.
Skin, diseases of, lectures on 14, 65, 147, 234, 278, 350, 401, 585, 539, 593, 657, anatomy of 14, 65, atrophy of 240, causes of organic 279, local 283, eitiology of 278, general diagnosis of 350, hemorrhage of 239, hypertrophy of 240, pathology of 234, prognosis of 358, secretion, disorders of 485, symptoms 147, therapeutics external and internal 403, objective 148, primary and secondary 151.
Snuff, the sale of in Atlanta 323.
Society reports: Atlanta Society of Medicine 172, 423, 619, Baltimore Academy of Medicine 38, 106, 306, 616, Chicago Medical Society 33, 90, 252, 302, 362, 425, 498, 555, 609, 690, Clinical Society of Maryland, 246, 369, Macon Medical Society 765, Sudamina 490, surgical notes 415.

T

The application of the intra-uterine pressure by the tampon to the cavity of the uterus in diseases of the endometrium 313, 465.

U

Ulcers of the lower leg, chronic 296.
Uridrosis 490.
Urithritis, chronic, 300.
Urticaria 597.
Uterine cavity, Vulliet's new method of dilating 242.
Uterine flexions, the treatment of 337, 641.

V

Vesical irritation in women 726.

Y

Yellow fever, commission and the national board of health 179, inoculation 581, inoculation *vs.* fools 449, regeneration of the virus of 717.

### THE MEDICAL ASSOCIATION OF GEORGIA.

The next meeting of the Association will be held in Augusta, Wednesday, Thursday and Friday, April 21, 22 and 23. From present indications it promises to be largely attended. Below we give the names of the officers and of the various committees who are expected to have their reports ready on the first day of the session:

*President*—R. J. Nunn, Savannah.

*First Vice-President*—L. B. Alexander, Forsyth.

*Second Vice-President*—T. F. Walker, Cochran.

*Committee on Publication*—James A. Gray, E. C. Goodrich, Robert Battey, H. V. M. Miller, W. D. Bizzell, J. P. Logan.

*Committee on Necrology*—K. P. Moore, A. G. Whitehead, G. W. Holmes, J. S. Todd.

*Committee on Prize Essays*—R. J. Nunn, Robert Battey, W. A. Love, H. F. Campbell, Eugene Foster.

*Committee of Arrangements*—E. C. Goodrich, J. M. Hull, C. W. Hickman, Eugene Foster, W. H. Doughty, jr., T. R. Wright.

SECTIONS—FIRST DISTRICT.

*Practice*—W. Duncan, K. A. Quarterman, J. J. Waring.
*Surgery*—W. H. Elliott, R. G. Norton, G. H. Stone.
*Gynecology*—R. B. Harris, E. W. Lane, J. Weichselbaum.

SECOND DISTRICT.

*Practice*—T. A. Chappell, W. B. Cheatham, L. B. Bouchelle.
*Surgery*—T. M. McIntosh, W. S. Wood.
*Gynecology*—A. P. Taylor, B. R. Doster, P. S. Hilsman.

THIRD DISTRICT.

*Practice*—A. A. Smith, Chas. Hicks, E. K. Bozeman.
*Surgery*—S. B. Hawkins, J. D. Herman, A. K. Taylor.
*Gynecology*—T. F. Walker, J. F. Fielder, R. O. Ingram.

FOURTH DISTRICT.

*Practice*—C. D. Smith, W. A. Pool, W. W. Fitts.
*Surgery*—J. T. Slaughter, J. A. Beasly, J. F. Cole.
*Gynecology*—A. W. Griggs, Geo. J. Grimes, Paul Faver.

FIFTH DISTRICT.

*Practice*—W. S. Elkin, W. H. Pool, Henry Gaither.
*Surgery*—James A. Gray, W. B. Parks, R. H. Taylor.
*Gynecology*—W. A. Love, J. McF. Gaston, T. S. Powell.

SIXTH DISTRICT.

*Practice*—C. H. Hall, J. C. Solomon, W. O'Daniel.
*Surgery*—Lewis Harris, H. McHatton, H. J. Williams.
*Gynecology*—K. P. Moore, T. O. Powell, L. B. Alexander.

SEVENTH DISTRICT.

*Practice*—T. M. Holmes, W. S. Kendrick, F. R. Calhoun.
*Surgery*—W. B. Wells, J. B. S. Holmes, E. H. Richardson.
*Gynecology*—Robert Battey, J. W. Clements, C. P. Gordon.

EIGHTH DISTRICT.

*Practice*—J. J. Hill, A. A. Bell, J. C. Pope.
*Surgery*—S. C. Benedict, E. D. Alfriend, R. B. Nisbet.
*Gynecology*—John Gerdine, A. Moody Burt, G. W. Mulligan.

NINTH DISTRICT.

*Practice*—J. B. Pendergrass, J. H. Goss.
*Surgery*—L. G. Hardman, W. J. Bush, R. L. Harris.
*Gynecology*—J. W. Bailey, W. A. Watson.

TENTH DISTRICT.

*Practice*—S. D. Brantley, A. H. Baker, J. M. Kelly.
*Surgery*—DeSaussure Ford, T. R. Wright, M. G. Hatch.
*Gynecology*—H. F. Campbell, W. H. Doughty, A. G. Whitehead.

### OUR ADVERTISERS.

SEE advertisement of the "Old Book Store" on page 26 and write for catalogue.

PEACOCK'S BROMIDES.—Dr. Jefferson Wilcox, of Hazlehurst, Ga., says: "I have used Peacock's bromides in my practice with success. A little girl twelve years old had been afflicted with epilepsy since she was three months old, having epileptic convulsions nearly every day, until I put her on Peacock's bromides. Since then she has not even had the symptom of one. It is surely a great remedy."

CELERINA.—Dr. B. F. Nicholls, of Philadelphia ("*Medical Brief*"), relates the histories of a number of cases in which he has used this preparation made by the Rio Chemical Company. From his experience with it, he believes it to be a remedy that will meet the indications in all cases where nervous prostration plays an important part. He has used it for nervous headache, nervous dyspepsia, spermatorrhœa, heart trouble dependent on disordered nervous action, and a number of other affections of like origin, and says that it has proved more satisfactory than any other remedy.

SCOTT'S EMULSION OF COD LIVER OIL.—Dr. T. S. Bell, Professor of State Medicine and Sanitary Science in the University of Louisville, says of this popular remedy: "I have been using Scott's Emulsion of Cod Liver Oil with Hypophosphites in my practice for several years, with more satisfaction growing out of success than any other preparation I have ever used. I commend it to my classes in the University of Louisville as much the best article of Cod Liver Oil."

D. H. FITCH, CAZENOVIA, N. Y.—Any of our readers in need of Galvanic or Faradic Batteries would do well to refer to the advertisement of this gentleman and write him for prices.

CANTON SURGICAL CHAIR.—We have been using one of these chairs in our office for several months, and we are entirely satisfied with it. It is superior to any surgical chair we know.

DR. J. T. JELKS, HOT SPRINGS, ARKANSAS, has a card in this issue of THE JOURNAL. Dr. Jelks was at one time a citizen of this State. He is at the head of the profession in Arkansas.

BRADFIELD & WARE, ATLANTA.—There is not a more reliable firm in the city than this one. Send to them for anything you may need in their line and you will be satisfied with what you get.

CHARLES O. TYNER, ATLANTA.—This gentleman comes to the front in this issue with a new advertisement that ought to be read by every one. We know Mr. Tyner personally, and can vouch for him in any business transaction.

ATTENTION is directed to the advertisement of the New York Post-Graduate School of Medicine on our third cover page. The faculty number among its members some of the most distinguished men in the profession in America.

REED & CARNRICK, NEW YORK.—These gentlemen have an attractive advertisement in this issue of THE JOURNAL. The different pharmaceutical preparations of this firm are all of the very best, and our readers may have no hesitancy in prescribing them for the various affections for which they are recommended.

COLDEN'S LIQUID BEEF TONIC.—This excellent preparation has become deservedly popular with the medical profession in the treatment of diseases where an agreeable article of diet and tonics required. It is recommended in typhoid and malarial fevers, consumption, loss of appetite and debility induced by any cause, and it is tolerated when other forms of animal food are rejected.—*Maryland Medical Journal.*

